# The Effect of Heat Shock on Seed Dormancy Release and Germination in Two Rare and Endangered *Astragalus* L. Species (Fabaceae)

**DOI:** 10.3390/plants13040484

**Published:** 2024-02-08

**Authors:** Alba Cuena Lombraña, Ludovica Dessì, Lina Podda, Mauro Fois, Belén Luna, Marco Porceddu, Gianluigi Bacchetta

**Affiliations:** 1 Centre for Conservation of Biodiversity (CCB), Sardinian Germplasm Bank (BG-SAR), Department of Life and Environmental Sciences, University of Cagliari, Viale Sant’Ignazio da Laconi 9-13, 09123 Cagliari, Italy; alba.cuena@unica.it (A.C.L.); lina.podda@unica.it (L.P.); mfois@unica.it (M.F.); porceddu.marco@unica.it (M.P.); bacchet@unica.it (G.B.); 2 Departamento de Ciencias Ambientales, Facultad de Ciencias Ambientales y Bioquímica, Universidad de Castilla-La Mancha, Av. Carlos III S/N, 45071 Toledo, Spain; belen.luna@uclm.es

**Keywords:** *Astragalus maritimus*, *Astragalus verrucosus*, fire, mediterranean vascular flora, physical dormancy, water imbibition

## Abstract

Many *Astragalus* species exhibit seeds with physical dormancy (PY), but little is known about the ecological context of this dormancy. We focused on *A. maritimus* and *A. verrucosus*, two threatened Sardinian endemic species inside the subgenus *Trimeniaeus* Bunge. Fresh seeds collected from the only two respective known populations were used to investigate the effect of mechanical scarification, heat shock, and water imbibition processes on PY release and germination. PY can be overcome through mechanical scarification of the water-impermeable seed coats, while no dormancy break was detected, nor a subsequent increase in seed germination due to fire-induced heat. This suggests that fire does not trigger dormancy release and seed germination in these species. The seeds tolerate relatively high heat shock temperatures (up to 120 and 100 °C for *A. verrucosus* and *A*. *maritimus*, respectively), but after 120 °C for 10 min, the number of dead seeds increases in both species. These facts suggest the capacity to develop a soil seed bank that can persist after fires and delay germination until the occurrence of optimal conditions. As regards water imbibition, both *Astragalus* species did not show the typical triphasic pattern, as germination started without further water uptake. This study emphasizes the significance of understanding germination processes and dormancy in threatened species. In fire-prone ecosystems, PY dormancy plays a crucial role in soil seed bank persistence, and it may be selectively influenced by post-fire conditions. Understanding such adaptations provides useful insights into conservation strategies.

## 1. Introduction

*Astragalus* L., belonging to the Fabaceae family, stands as the largest genus among plants, encompassing over 3000 species distributed naturally across multiple continents [1,2,3]. This genus holds significance in forage, medicinal, and industrial applications. However, certain species within *Astragalus* are classified as rare and endemic, and some of them are threatened by extinction. Notably, a considerable proportion of *Astragalus* species exhibit physical dormancy (PY) [3]. The seeds of these species are known to be dormant at maturity, necessitating the exploration of different treatments to break this dormancy and promote germination. Considering the huge size of the genus, seed dormancy has been studied in relatively few species of *Astragalus*, most of which are perennials. However, the literature on seed dormancy in perennial and annual *taxa* reports all of them to have water-impermeable seeds and thus show PY [4]. PY, which is due to a water-impermeable seed coat, occurs in the seeds of 18 plant families of Angiospermae, including the Fabaceae [5,6,7].

The proportion of seeds in a particular species that develops impermeable coats can vary based on the environmental conditions of the mother plant during seed development, the degree of drying after the seed is fully developed, and genetic factors [8]. It is thus important to investigate the imbibition process and the breaking of dormancy before studying other aspects of seed ecophysiology or their ecological implications.

Impermeable seed coats can avoid early imbibition and shift seed germination to a more appropriate time for seedling recruitment [9,10,11]. In addition, these types of coats may contribute to protecting embryos from the harmful effects of the high temperatures reached during a fire event [12,13]. Indeed, fire can significantly impact the reproductive capacity of plants by affecting the soil seed bank [14,15,16,17]. It can accelerate seed germination of some species but also cause seed mortality of many others [18], with effects dependent on seed tolerance to the high temperatures experienced during a fire event.

Fire-prone ecosystems serve as hotspots of seed dormancy, allowing seed banks to accumulate in both the soil and the canopy [19,20,21]. Within these ecosystems, physical dormancy can be broken in several species by the heat shocks generated during wildfires [8,22], allowing seed imbibition and germination [23,24]. Determining how high temperatures interfere with the physical barriers for germination contributes to unraveling which ones are effectively heat-tolerant and how frequent fires affect their recruitment patterns [11,22]. Furthermore, it is generally considered that in species with physical dormancy (PY), once the seeds become permeable to water, germination occurs over a broad range of temperatures [10]. However, it is important to note that this aspect requires further investigation across various taxa. This information is essential to improve our understanding about the impacts of fire on vegetation resilience and to direct policies focused on long-term conservation of native vegetation [11,25].

Thriving in the warmest and driest environments of Europe, the Mediterranean vegetation type is considered highly vulnerable to climate-change impacts, primarily due to aridity, disturbance-driven forest decline, biodiversity loss, and changes in vegetation, which also increase the frequency of wildfires [26] and references therein. In this context, heat-shock-stimulated germination is prominently observed in Mediterranean-type ecosystems of California, the Mediterranean Basin, South Africa, and southwestern Australia [16,27,28,29,30]. Heat-shock-stimulated germination is also a phylogenetically conserved trait [31] and is therefore restricted to a few families [32]. In the Mediterranean Basin, for example, heat-shock-stimulated germination is mainly observed in the Fabaceae and Cistaceae [33] and references therein. To better understand these species’ response to heat shock treatments, we followed the classification proposed by Luna et al. [34]. This classification divides species into three main categories: (I) species stimulated by heat shock (those species that increase their germination under any of the three heat shock treatments with respect to the control); (II) species that are tolerant of but not stimulated by heat shock (species that do not show differences among heat shock treatments); and (III) non-tolerant species (these species suffer a germination decrease with any heat shock treatment).

*Astragalus verrucosus* Moris and *A. maritimus* Moris are two endemic species exclusive to Southwest Sardinia (Italy) that live in xeric and fire-prone environments. Previous germination studies [35] on these two species demonstrated that both have a germination inhibition due to tegument impermeability (PY) that is strongly dependent on the imbibition percentage. The same authors [35] confirmed that the two taxa did not show a physiological component of dormancy, and seeds germinated without problems after imbibition. However, the imbibition process and the effect of heat shock were, to our knowledge, never tested for these species. Structural studies in seeds of *A. maritimus* and *A. verrucosus*, or in seeds with PY more generally, are important to better understand their causes and release when subjected to treatments for dormancy breaking [36].

We hypothesized that fire-prone ecosystems create ideal conditions for the selection of seed dormancy as fire provides a mechanism for dormancy release and post-fire conditions are optimal for germination. To explore this hypothesis, we addressed the following main aims: (I) to explore the effect of different temperatures of heat shock on breaking dormancy and promoting germination and (II) to determine the post-fire strategies of these species. To achieve these objectives, we conducted controlled laboratory experiments that simulated diverse fire conditions through the application of heat shock treatments. Additionally, we evaluated (III) the seeds’ water imbibition capacity and (IV) the influence of scarification on broken physical dormancy (PY).

## 2. Results

### 2.1. Imbibition of Water and Germination Trials

**Figure 1:**
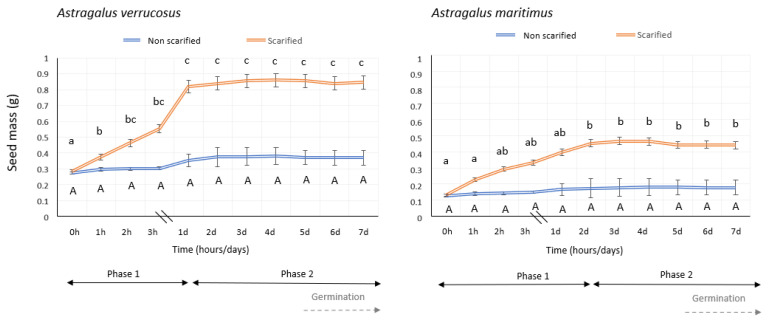
Imbibition of water of both *Astragalus* non-scarified and scarified seeds during the first week. Post hoc pairwise *t* test comparisons were carried out for the variations in seed mass across the time, and bars with different letters indicate significant differences (*p* < 0.05). Capital letters for non-scarified seeds and lowercase letters for scarified seeds. The beginning of germination is indicated with a dashed arrow.

The initial seed mass, considered as the mean seed mass of the population, was 0.0107 ± 0.0010 g in the case of *A. verrucosus* and 0.0059 ± 0.0010 g in the case of *A. maritimus*. The two investigated species showed significant differences (*p* < 0.001, Table 1) among themselves as a response to an increase in the mass depending on the treatment. When considering the variations in seed mass between scarified and non-scarified seeds in both species, significant differences (*p* < 0.001) were observed during the imbibition process (Figure 1 and Table 1). Scarified seeds of *A. verrucosus* showed a rapid initial mass gain during the first day (phase 1), followed by minimal variation in seed mass over the next five days (phase 2). The mass values of scarified seeds tended to stabilize during phase 2, which continued until the end of the imbibition process (day 5), when germination occurred, resulting in a regain of seed fresh mass. In scarified seeds of *A. maritimus*, the water content increased, whereas non-scarified seeds showed a lower increase in mass throughout the entire duration. In scarified *A. maritimus* seeds, there was an initial increase in seed mass within the first hour, which continued for two days (phase 1). After this period, scarified seeds remained at a constant mass for six more days (phase 2), leading to germination on the fifth day (Figure 1).

*Astragalus verrucosus* seeds nearly doubled their initial mass after one day, whereas *A. maritimus* required two days to enter phase 2. In terms of the increase in seed mass, scarified *A. verrucosus* seeds exhibited a 207.49% increase after one day compared to the 29.03% increase in non-scarified seeds, reaching their maximum mass on the third day (scarified, 262.02%). In the case of *A. maritimus*, after one day of imbibition, scarified seeds increased their mass by 196.74%, compared to the 29.04% increase in non-scarified seeds during the same period. The maximum increase in seed mass for scarified *A. maritimus* seeds was reached on the fourth day, with a 211.57% increase.

**Figure 2:**
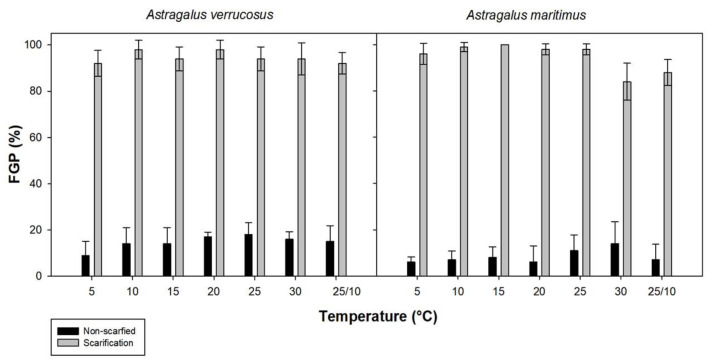
Final Germination Percentage (FGP) of non-scarified and scarified seeds from both *Astragalus* species under different temperature conditions (5, 10, 15, 20, 25, 30, and 25/10 °C; the latter temperature corresponds to an alternating regime with a day/night cycle: 12 h light/12 h dark). The data represent the mean of four replicates (±SD).

For *A. verrucosus* and *A. maritimus*, the mechanical scarification treatment had a significant impact on germination outcomes (*p* < 0.001, Table 2 and Figure 2), and no differences between species and incubation temperature factors were highlighted by the analysis (*p* = 0.208 and *p* = 0.119, respectively, Table 2). The interaction between treatment and temperature was statistically significant (*p* = 0.009). This implies that the combined effect of treatment and temperature did significantly impact on the Final Germination Percentage (FGP). Other interactions were less statistically significant, such as the effect of treatment by species (*p* = 0.030), because the *A. maritimus* germination percentage in non-scarified seeds was lower than that for *A. verrucosus*, and temperature by species (*p* = 0.324). In both species, scarification treatment had a substantial impact on germination (more than 80%, independent of the incubation temperature, Figure 2), significantly improving the FGP. In *A. verrucosus*, the highest percentage of germination was recorded at 10 and 20 °C (98%). Similarly, in *A. maritimus*, 100% germination was achieved at a temperature of 15 °C, and germination percentages of 98–99% were also achieved at temperatures of 10, 20, 25, and 30 °C.

### 2.2. Heat Shock (HS)

**Figure 3:**
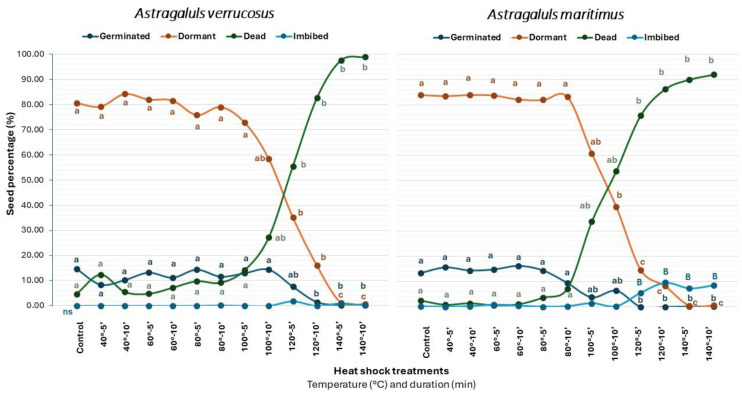
Percentages (%) of germinated, dead, dormant, and imbibed seeds of *A. verrucosus* and *A. maritimus* under control (non-scarified) and different heat shock (HS) treatments and durations (five or ten minutes). Statistical comparisons were conducted using post hoc pairwise *t*-tests, and values with the same letter are not considered statistically different at *p* > 0.05. Letters in different colors indicate data from distinct seed categorizations at the conclusion of the experiments. Capital letters were used for imbibed seeds.

After a preliminary data analysis, it was observed that incubation temperatures did not present statistically significant differences regarding the FGP. Consequently, we opted to combine germination data obtained from different HS treatments, independent of the incubation temperatures. This approach facilitated a comprehensive examination of dry heat shock conditions (Figure 3). Both *Astragalus* species did not present differences in germination across the different dry heat shock treatments (*p* = 0.111, Table 3). However, the interaction between species and treatment was significant (*p* < 0.05, Table 3), showing a small difference in the reduction in germinated seeds at higher temperatures of HS in *A. verrucosus* (120 °C for five minutes) versus *A. maritimus* (100 °C for ten minutes). Different conditions of HS had also a significant effect on the subsequent percentage of dead and dormant seeds (*p* < 0.001, Table 3 and Figure 3). In the case of *A. verrucosus*, the effect of HS was significantly different at 120 °C for ten minutes, resulting in an increment in the percentage of dead seed (*p* < 0.05, Figure 3). In the case of *A. maritimus*, significant differences in the number of dead seeds were found in the treatment of 120 °C for five minutes (Figure 3). Instead, the other HS treatments showed similar results to the control in both species (*p* < 0.05). While lower HS treatments (40–100 °C) had no effect on germinated, dead, dormant, or imbibed seeds, the hottest treatments (120–140 °C) produced a significant negative effect in these species (Figure 3). The percentage of imbibed seeds under different HS conditions was not statistically different in *A. verrucosus*. In contrast, for *A. maritimus*, exposure to HS at temperatures exceeding 120 °C for ten minutes resulted in an increase in imbibed seeds (*p* < 0.05), indicating that the impermeability of the tegument was broken in 10% of the total tested seeds.

## 3. Discussion

The water imbibition process generally occurs with an initially rapid imbibition because of the water potential gradient between the dry seed and external environment (phase I). Simultaneously, the metabolic activity of the seed is restored, which characterizes the beginning of the germination process. This stage is followed by a period of limited water absorption (phase II) and is the final stage for dead or dormant seeds. The last stage (phase III) is characterized by renewed hydration that culminates in the emission of a radicle and finalization of germination [37]. The triphasic pattern during seed imbibition proposed by Bewley and Black [38] seems not to be a general rule [39]; other studies have failed to identify a triphasic pattern of seed imbibition. For example, within the Fabaceae family, no triphasic pattern was found in *Lupinus albus* L. seeds when the data were expressed by water content [40], and the same was observed in *Glycine max* (L.) Merr. seeds [41]. A similar behavior occurs among other families, for example, *Cucumis anguria* L. [42] and *Brachiaria brizantha* (A.Rich.) Stapf [43]. All these cases relied on the seed water content to define the imbibition curve. Therefore, the standard triphasic pattern was not observed in the two investigated *Astragalus* species, as no renewed hydration was observed in either of them in phase 3; the seed water content stabilized during phase 2 and coincided with germination, which began on the fifth day and concluded two days later.

The results obtained with the imbibition test carried out in scarified and non-scarified seeds agree with those obtained for other *Astragalus* species (e.g., *A. arpilobus* Kar. & Kir. and *A. nitidiflorus* Jiménez & Pau) in which most of the seeds are dormant due to a water-impermeable seed coat [4,35,44], making scarification necessary to interrupt the physical dormancy (PY). In this study, we detected that non-scarified seeds of both species had a much lower imbibition capacity than did scarified seeds.

The germination experiments showed a positive effect of mechanical scarification on seed germination, in agreement with results obtained for *Astragalus maritimus* and *A. verrucosus* [35]. This method increased the percentage of germinating seeds to near 100% in both species, independently of the incubation temperature. Thus, mechanical scarification turned out to be the best method of breaking dormancy, and these results confirmed the assumptions that *A. verrucosus* and *A. maritiumus* seeds have a water-impermeable seed coat and can be included in the group of seeds characterized by PY, according to the classification proposed by Baskin and Baskin [8].

Incubation temperatures had no effect on seed germination in non-scarified, scarified, and heat-shock-treated seeds. These species show a wide thermal germination niche since both were able to germinate under a wide range of temperatures. Additionally, once PY was broken, germination rates were very high at the different incubation temperatures, which suggests that germination is determined by the moment at which dormancy is broken, regardless of the temperature.

Considering the effect of heat shock on breaking PY and promoting germination, a reduced germination rate compared with that in the control test was observed only at high heat shock temperatures in both species. *Astragalus verrucosus* tolerates heat shock (less than 50% dead seeds) until temperatures of 120 °C for five minutes, while *A. maritimus* tolerates heat shock until temperatures of 100 °C for ten minutes. At higher temperatures of heat shock, the percentage of dead seeds increases considerably. In the case of *A. verrucosus*, the percentage of dead seeds ranged from 55.4% at 120 °C for five minutes to 99% at 140 °C for ten minutes. For *A. maritimus,* this value varied from 54% at 100 °C for ten minutes to 92% dead seeds at 140 °C for five minutes.

In agreement with Kazancı and Tavşanoğlu [30], seeds of species belonging to families with PY (i.e., Fabaceae and Cistaceae) resisted even high heat shock temperatures. Our results also corroborate the findings of a study that included many Mediterranean plant species [31]. However, we did not find enhanced germination or dormancy break after moderate to high heat shocks in this species. Heat shock caused by fire is likely not able to break the PY in the investigated *Astragalus* seeds, which is imposed by impermeable seed coats. The germination rates of both species at different heat shock temperatures were similar to those of the control until the temperature of 120 °C, while heat shock at higher temperatures killed the seeds of both species. Considering the percentage of seeds that remained dormant (i.e., viable, not-imbibed seed) after the simulation of fire, for *A. verrucosus*, this percentage decreased from 80% at 40 °C for five minutes to less than 20% at 120 °C for ten minutes. In *A. maritimus*, the percentage of dormant seeds after the heat shock ranged from near 85% at 40 °C for five minutes to less than 10% at 120 °C for ten minutes. The percentage of dead seeds started to be significant after 120 °C for 5 min for *A. verrucosus* and after 100 °C for 10 min for *A maritimus*. At these temperatures and durations, we can indicate the fire tolerance limit for the species under study. *Astragalus maritimus* seems to be slightly more sensitive to the high-temperature conditions and consequences of fire passage than *A. verrucosus*. In summary, both species showed similar behavior under heat shock treatments, although the tolerance to high temperature appears to be slightly higher in *A. verrucosus*. Both species have the capacity to develop a soil seed bank able to persist after fires and delay germination until the occurrence of optimal conditions. This is a common process that leads to germination and seedling establishment in post-fire environments [34]. Considering the species’ responses to heat shock treatment (with germination percentages around 5–15% of the FGP) following the classification proposed by Luna et al. [34], both *Astragalus* can be included in category II, being tolerant of but not stimulated by heat shock.

In addition, considering that a small proportion of seeds germinated after low and moderate heat shock, both *Astragalus* species can be categorized as “facultative seeders”, which refers to plants capable of resprouting and germinating after a fire, following the post-fire regeneration strategies proposed by Pausas and Lamont [9]. Generally, it is widely accepted that fire-induced dormancy syndromes are prominent, if not dominant, in many fire-prone ecosystems [9]. However, within some of these ecosystems, certain species may possess soil-stored seed banks that do not respond to either heat or smoke, despite their seeds’ ability to withstand fire, similar to that of species with fire-released dormancy. This phenomenon is referred to as “non-fire-released dormancy” [9]. In such cases, dormancy release does not occur immediately after a fire but is induced by a variety of environmental stimuli, which may sometimes include an indirect role for fire or any other disturbance that opens up the vegetation. Further research is necessary to understand the environmental conditions that maintain and release imposed dormancy (once inherent dormancy is released or does not apply), as changes in climate cues could be key to plant success under a changing climate [9].

After analyzing the post-fire strategies of *A. verrucosus* and *A. maritimus* and considering findings from a previous study by Bacchetta et al. [35], which demonstrated that acid scarification (30 min bath in H_2_SO_4_) significantly increased germination rates for *A. verrucosus* (approximately 88%) and *A. maritimus* (around 81%), though with slightly lesser efficacy compared to mechanical scarification, a plausible hypothesis emerges regarding the endozoochory of these species. It has been established in other *Astragalus* species, as seen in *A. adsurgens*, that physical dormancy can be broken by treating seeds with HCl, particularly after undergoing gut passage [44]. Endozoochory is considered a key ecological and evolutionary process that provides significant benefits to numerous PY species worldwide because the dormancy of many seeds is broken, and seeds are moved away from the parent plant [45]. However, further studies are needed to better understand whether endozoochory is effectively able to break PY in *A. verrucosus* and *A*. *maritimus*.

## 4. Materials and Methods

### 4.1. Study Species, Field Site Description, and Seed Collection

*Astragalus verrucosus* is a perennial plant with a prostrate habit. It flowers between March and June and bears fruit between May and August. It is characteristic of hemicryptophytic grassland communities belonging to the association *Stipo bromoidis–Astragaletum verrucosi* Bacch., Brullo, Giusso, & Guarino [46]. It is a narrow Sardinian endemic species, only one population is known in the territory of Arbus (southwestern Sardinia). It is listed in the IUCN as Critically Endangered (CR); the main threats were identified in tourism development and land-use changes [35].

*Astragalus maritimus* is a cespitose hemicryptophyte. It flowers from April until the first half of May and bears fruit between April and May. It is a narrow endemism of Sardinia, and only one population is known in the coastal area of Cala dello Spalmatore on the Island of San Pietro (Carloforte). It grows in *Juniperus turbinata* Guss. shrublands and coastal rocky places. It is included in the IUCN Red Lists as Critically Endangered (CR), as it is mainly threatened by the passage of vehicles and motorbikes [35]. Both are listed as priority taxa in Annexes II and IV of the ‘Habitats Directive’ 92/43/EEC [47]. In both species, seed collection followed the internationally recognized protocols in order to maximize the genetic diversity and preserve the natural population by collecting < 30% of available seeds from the maximum number of individuals [48,49,50].

Mature pods of *A. verrucosus* and *A. maritimus* were collected during summer 2022 in natural populations. Seeds were manually extracted from the pods in the laboratory. All accessions were stored under controlled conditions (20 °C and 40% relative humidity) [48,49] for two weeks at the Sardinian Germplasm Bank (BG-SAR) before the germination tests [51].

### 4.2. Imbibition of Water and Germination Trials

To determine whether the seed coat is permeable or impermeable to water, the capacity for imbibition of water was compared in scarified and non-scarified seeds through an evaluation of the seed mass increase over time. Seeds of both species were scarified individually with a scalpel (mechanical scarification), and four replicates of 25 scarified and non-scarified seeds each were used and put inside beakers containing deionized water for a total of seven days. At time zero and at one-hour intervals for the first three hours, followed by one-day intervals for seven days, seeds were removed from the water and weighed; after this, seeds were again placed in the beakers containing water. For each replicate of scarified and non-scarified seeds, the percentage increase in seed mass was calculated using the following equation [8,25]: % increase in mass = $\frac{(Wi-Wd)\;}{Wd}\times 100$, where Wi = mass of imbibed seeds and Wd = mass of dry seeds.

Two seed treatments, intact and mechanical scarification, were chosen to carry out the germination test for evaluating dormancy-breaking requirements. In the mechanical scarification, a small portion of the seed coat was clipped manually with a scalpel, and care was taken to avoid damaging the cotyledon. Concretely, four replicates of 25 randomly selected seeds of both species were sown in 90 mm diameter plastic Petri dishes for each experimental condition with 1% agar water substrate and incubated with a day/night cycle (12 h light/12 h dark) at 5, 10, 15, 20, 25, 30, and 25/10 °C (alternating temperature), in germination chambers (MLR-351, Sanyo, Tokyo, Japan) with white fluorescent lamps (FL40SS.W/37 70–10 μmol m^−2^ s^−1^, Sanyo, Tokyo, Japan). The germination was checked three times a week, and germinated seeds were scored when a radicle ≥1 mm long was visible. After a minimum period of 90 days, if no additional germination had occurred for two further weeks, non-germinated seeds were cut with a scalpel to determine the number of imbibed or filled, not imbibed but viable (dormant), dead, and empty seeds [48,49,51].

### 4.3. Heat Shock (HS)

Fresh seeds were exposed to 40, 60, 80, 100, 120, and 140 °C in a drying oven for 5 and 10 min in order to simulate the temperature–time curves identified during experimental fires over shrublands [34]. Seeds were then sown in 90 mm diameter plastic Petri dishes for each experimental condition with 1% agar water substrate and incubated with a day/night cycle (12 h light/12 h dark) at 15, 20, 25, 30, and 25/10 °C (alternating temperature) for 90 days, following the methodology of [25]. Afterward, non-germinated seeds were cut with a scalpel to determine the number of imbibed or filled, not imbibed but viable (dormant), dead, and empty seeds [48,49,51]. For each treatment of dry heat shock and temperature, we used four replicates of 25 seeds.

### 4.4. Data Analysis

The Final Germination Percentage (FGP) was calculated as the mean of the four replicates (±SD) based on the total number of filled seeds (empty seeds were excluded). Generalized Linear Models (GLMs) were used (I) to evaluate differences in the increment in seed mass between scarified and non-scarified seeds over time; (II) to evaluate the effect of mechanical scarification and incubation temperatures on the FGP in both species; and (III) to evaluate the effect of different heat shock treatments on the percentages of germinated, dormant, imbibed, and dead seeds in both species. Quasi-Poisson and quasi-binomial error structures, the latter with the logit link function and *F*-tests with an empirical scale parameter instead of chi-squared on the subsequent ANOVA, were used to overcome residual overdispersion [52]. Significant differences highlighted by GLMs (<0.001 were then analyzed by a post hoc pairwise-comparison *t*-test (with Bonferroni adjustment). All statistical analyses used R v. 3.0.3 [53].

## 5. Conclusions

High heat shock temperatures did not enhance germination in the investigated *Astragalus* species; instead, the seeds remained dormant. This observation demonstrates the tolerance of seeds with physical dormancy to fire-induced heat. Ecologically, this indicates that even after a fire event, seeds can remain stored in the soil until dormancy is broken by specific environmental cues, such as scarification. This process has crucial ecological implications for post-fire recovery and persistence in fire-prone ecosystems, highlighting the interplay between fire, dormancy, and regeneration strategies in these ecosystems. In addition, the variability in seed imbibition patterns has ecological implications, highlighting the need for a nuanced understanding of germination processes in different species and ecosystems. Mechanical scarification is highly effective for breaking seed dormancy in *A. verrucosus* and *A. maritimus,* confirming the presence of physical dormancy. These findings point to the ecological diversity and adaptation of plant species to their specific environments, emphasizing the need for tailored conservation and management strategies. For instance, narrow endemic species in fire-prone Mediterranean ecosystems, such as those here studied, have survived and adapted to particular environments favored by fires, where seed dormancy has allowed these species to compete with dominant plant communities and take advantage of bare patches of vegetation created by fires for seedling growth.

## Figures and Tables

**Table 1:** GLM results of seed mass (log transformed) during the imbibition period, depending on the following factors: treatment (non-scarified and scarified), time (0, 1, 2, and 3 h refer to the three first hours of water imbibition and 1–7 d refers to the time in days), species (*A. verrucosus* and *A. maritimus* seeds), and their interactions.

Coefficients:	Estimate	Std. Error	*t* Value	*p* Value
(Intercept)	−1.743	0.103	−16.813	<0.001 ***
Treatment	0.934	0.131	7.148	<0.001 ***
Time	0.028	0.017	1.601	0.11
Species	0.973	0.132	7.429	<0.001 ***
Treatment x Time	0.051	0.022	2.344	0.019 *
Treatment x Species	0.009	0.173	0.053	0.957
Time x Species	0.005	0.022	0.231	0.817
Treatment x Time x Species	0.134	0.031	4.283	<0.001 ***

Signif. codes: *** *p* < 0.001; * *p* < 0.05.

**Table 2:** GLM results of Final Germination Percentage (FGP) in *A. verrucosus* and *A. maritimus* seeds depending on the following factors: treatment (non-scarified and scarified), incubation temperature (5, 10, 15, 20, 25, 30, and 25/10 °C), species, and their interactions.

	Estimate	Std. Error	*t* Value	*p* Value
(Intercept)	−280.185	0.596	−4.696	<0.001 ***
Treatment	1048.816	197.371	5.314	<0.001 ***
Temperature	0.039	0.024	1.582	0.119
Species	100.929	0.793	1.272	0.208
Treatment x Temperature	−0.197	0.074	−2.671	0.009 **
Treatment x Species	−495.658	222.635	−2.226	0.030 *
Temperature x Species	−0.033	0.033	−0.995	0.324
Treatment x Temperature x Species	0.152	0.085	1.787	0.079

Signif. codes: *** *p* < 0.001; ** *p* < 0.01; * *p* < 0.05.

**Table 3:** GLM results of percentage of germinated, dead, dormant, and imbibed seeds in *A. verrucosus* and *A. maritimus* seeds depending on the following factors: treatment (non-scarified) and different heat shock (HS) treatments with different durations (five or ten minutes), species, and their interactions.

	*F*-Tests	Df	Sum Sq	Mean Sq	F	*p* Value
**Germinated Percentage**	Treatment	12	1.585	0.132	36.137	0.001 ***
	Species	1	0.009	0.009	2.546	0.111
	Species x Treatment	12	0.301	0.251	6.867	<0.05 *
**Dead Percentage**	Treatment	12	67.730	5.644	678.256	<0.001***
	Species	1	0.020	0.024	2.929	0.680
	Treatment x Species	12	2.450	0.204	24.503	<0.05 *
**Dormant Percentage**	Treatment	12	55.050	4.587	613.39	<0.001***
	Species	1	0.130	0.128	17.12	<0.05 *
	Treatment x Species	12	1.220	0.101	13.55	<0.05 *
**Imbibed Percentage**	Treatment	12	0.189	0.015	6.34	<0.05 *
	Species	1	0.074	0.074	30.115	<0.05 *
	Treatment x Species	12	0.146	0.012	4.869	<0.05 *

Signif. codes: *** *p* <0.001; * *p* < 0.05.

## Data Availability

Data are contained within the article.
